# Supplementary material for: A Longitudinal Study of Exposure to Manganese and Incidence of Metabolic Syndrome
Source: Nutrients. 2022 Oct 13;14(20):4271. doi: 10.3390/nu14204271 (PMC9607173; doi:10.3390/nu14204271)
Supplement: Supplementary file 1 [file nutrients-14-04271-s001.zip › nutrients-1939607-supplementary.pdf]

Supplemental Table S1. Distributions [a] of metals (μg/L) at baseline among participants included in the analysis (n = 1478), contrasted by the median Mn value

	All	Mn ≤ 0.63	Mn > 0.63	n (%) below the limit of detection
Arsenic	16.12 (7.12, 32.88)	14.84 (5.95, 31.84)	16.70 (8.25, 34.38)	109 (7.4)
Barium	2.16 (1.23, 4.27)	1.62 (0.93, 3.10)	2.98 (1.69, 5.69)	20 (1.4)
Cadmium	0.66 (0.37, 1.20)	0.52 (0.29, 0.94)	0.85 (0.51, 1.48)	69 (4.7)
Cobalt	0.55 (0.33, 0.93)	0.47 (0.26, 0.80)	0.62 (0.41, 1.07)	25 (1.7)
Chromium	0.44 (0.40, 1.51)	0.40 (0.38, 0.80)	0.59 (0.40, 2.33)	444 (30.0)
Cesium	3.37 (2.09, 5.11)	2.63 (1.71, 4.08)	4.14 (2.79, 6.05)	1 (0.1)
Copper	22.85 (13.20, 46.00)	14.61 (9.6, 22.59)	41.80 (23.62, 67.50)	11 (0.7)
Molybdenum	36.69 (21.50, 58.01)	27.23 (16.75, 44.92)	47.35 (30.70, 69.25)	6 (0.4)
Lead	2.70 (1.42, 7.46)	1.91 (1.09, 5.24)	3.73 (2.02, 10.60)	22 (1.5)
Selenium	37.66 (22.29, 57.27)	29.88 (17.63, 46.43)	47.12 (31.60, 66.25)	2 (0.1)
Thallium	0.10 (0.06, 0.17)	0.08 (0.06, 0.12)	0.14 (0.09, 0.21)	317 (21.5)
Tungsten	0.22 (0.20, 0.60)	0.20 (0.18, 0.65)	0.24 (0.20, 0.55)	463 (31.3)
Zinc	452.38 (256.25, 736.60)	341.45 (191.53, 602.46)	543.50 (345.21, 858.30)	0 (0.0)

[a] Metal values are median (25th, 75th percentiles) for the metal listed in the row

Supplemental Figure S1. Exposure-response functions of natural log-transformed (ln) urinary metal concentrations and odds estimates of metabolic syndrome (MetS) (n = 1478)

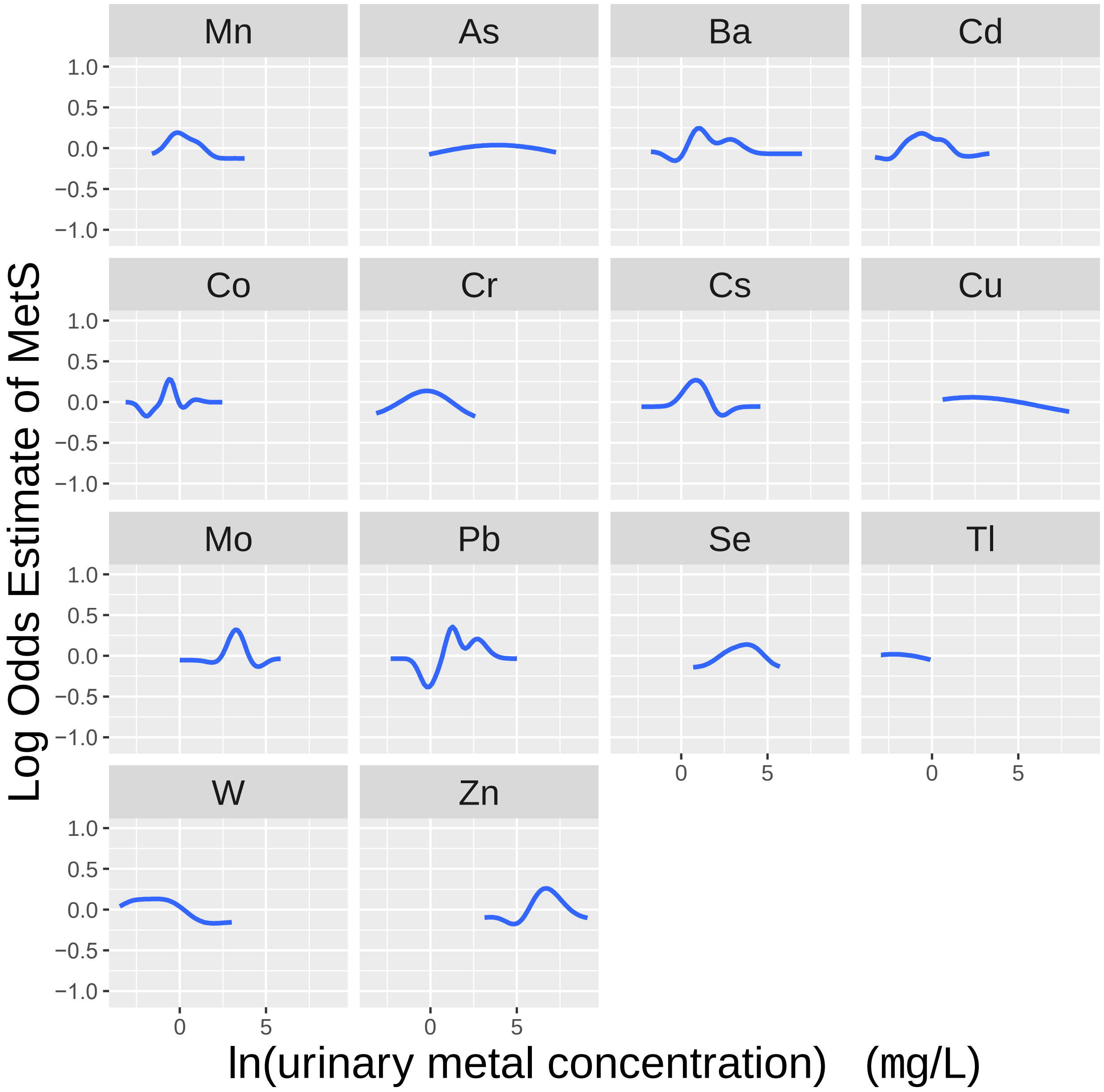

Models were adjusted for baseline values of sex, age (years), ethnicity (Hispanic, non-Hispanic), annual gross household income (<$10,000, $10,000-$24,999, ≥$25,000), smoking status (<100 cigarettes in lifetime (never), ≥100 cigarettes in lifetime and does not currently smoker (former), ≥100 cigarettes in lifetime and currently smokes (current)), caloric intake (kcal/day), and urinary creatinine (g/L). Metabolic syndrome was defined as having three or more of the following outcomes: 1) waist-hip ratio (waist circumference divided by iliac circumference) >0.90 for males, waist-hip ratio >0.85 for females, or a body mass index (measured weight divided by measured height squared) >30 kg/m^2, 2) high density lipoprotein <40 mg/dL for males or <50 mg/dL for females, 3) triglycerides ≥150 mg/dL, 4) fasting glucose ≥100 mg/dL or diabetes (includes people who self-reported being diagnosed with diabetes via an oral glucose tolerance test or prescribed insulin or oral hyperglycemic medication), or 5) measured systolic blood pressure ≥130 mmHg or diastolic blood pressure ≥85 mmHg or self-reported current use of antihypertensive medications.

Supplemental Table S2. Crude longitudinal associations [a] between baseline quartiles of manganese and metabolic outcomes

	Quartile 2<br>0.33–0.63 µg/L	Quartile 3<br>0.63–1.13 µg/L	Quartile 4<br>1.13–42.5 µg/L	*p-value for trend*
Waist-hip ratio; *n = 1477*	−0.013 (−0.022, −0.004)	−0.013 (−0.023, −0.004)	−0.015 (−0.024, −0.005)	<0.01*
Males	0.002 (−0.005, 0.010)	0.000 (−0.007, 0.007)	−0.001 (−0.007, 0.006)	0.69
Females	−0.003 (−0.012, 0.006)	−0.003 (−0.013, 0.006)	−0.001 (−0.011, 0.009)	0.84
Body mass index (kg/m²); *n = 1477*	−0.06 (−0.74, 0.63)	0.23 (−0.47, 0.93)	0.45 (−0.26, 1.16)	0.16
Males	−0.03 (−0.88, 0.81)	−0.51 (−1.34, 0.31)	−0.21 (−1.03, 0.61)	0.43
Females	−0.10 (−1.16, 0.96)	0.85 (−0.27, 1.97)	1.04 (−0.14, 2.22)	0.03*
High-density lipoprotein (mg/dL); *n = 1476*	0.03 (−1.80, 1.85)	−0.25 (−2.11, 1.62)	0.85 (−1.05, 2.75)	0.46
Males	−0.83 (−3.14, 1.49)	−0.26 (−2.52, 1.99)	0.14 (−2.11, 2.38)	0.80
Females	−1.39 (−3.98, 1.19)	−2.18 (−4.92, 0.55)	−0.80 (−3.68, 2.08)	0.49
Triglycerides (mg/dL); *n = 1477*	−12.3 (−29.8, 5.2)	−7.5 (−25.4, 10.4)	−14.0 (−32.2, 4.2)	0.21
Males	−3.0 (−23.5, 17.5)	−8.9 (−28.8, 11.1)	−19.3 (−39.1, 0.5)	0.047*
Females	−21.3 (−50.6, 8.0)	−7.6 (−38.7, 23.5)	−9.0 (−41.7, 23.8)	0.83
Fasting glucose (mg/dL); *n = 1475*	−0.2 (−7.7, 7.3)	−7.2 (−14.9, 0.5)	−10.1 (−17.9, −2.3)	<0.01*
Males	5.6 (−4.7, 15.9)	−11.9 (−22.0, −1.9)	−13.7 (−23.7, −3.7)	<0.01*
Females	−1.2 (−12.1, 9.6)	−0.2 (−11.7, 11.3)	−3.6 (−15.6, 8.5)	0.63
Systolic blood pressure (mmHg); *n = 1477*	−2.9 (−5.5, −0.3)	0.3 (−2.3, 3.0)	0.3 (−2.4, 3.0)	0.37
Males	0.0 (−3.5, 3.4)	−2.6 (−6.0, 0.8)	−0.6 (−3.9, 2.8)	0.49
Females	−4.0 (−7.9, −0.2)	4.1 (0.0, 8.1)	2.4 (−1.9, 6.6)	0.02*
Diastolic blood pressure (mmHg); *n = 1477*	−1.7 (−2.9, −0.5)	−1.4 (−2.6, −0.2)	−0.9 (−2.1, 0.3)	0.23
Males	−0.8 (−2.5, 0.9)	−1.8 (−3.5, −0.2)	−0.8 (−2.4, 0.9)	0.25
Females	−1.3 (−2.9, 0.2)	0.0 (−1.6, 1.7)	0.1 (−1.6, 1.9)	0.48

*p<0.05

[a] Associations assessed using linear mixed effects models with a random intercept for each participant. The reference group for manganese is the first quartile (≤ 0.33 µg/L).

Supplemental Table S3. Adjusted longitudinal associations [a] between baseline quartiles of manganese and metabolic outcomes among participants with Mn levels above the limit of detection

	**Quartile 2** 0.33–0.63 µg/L	**Quartile 3** 0.63–1.13 µg/L	**Quartile 4** 1.13–42.5 µg/L	***p-value for trend***
Waist-hip ratio; *n = 1235*	0.000 (−0.008, 0.008)	−0.002 (−0.010, 0.006)	0.000 (−0.008, 0.008)	0.99
Males	0.003 (−0.007, 0.014)	0.001 (−0.009, 0.012)	0.001 (−0.010, 0.011)	0.77
Females	−0.002 (−0.014, 0.009)	−0.002 (−0.013, 0.010)	0.002 (−0.010, 0.014)	0.54
Body mass index (kg/m^2); *n = 1235*	−0.10 (−1.06, 0.86)	−0.01 (−0.99, 0.96)	0.23 (−0.76, 1.22)	0.46
Males	−0.37 (−1.62, 0.88)	−1.05 (−2.30, 0.19)	−0.55 (−1.79, 0.69)	0.38
Females	0.09 (−1.30, 1.48)	0.71 (−0.73, 2.15)	0.90 (−0.61, 2.40)	0.12
High-density lipoprotein (mg/dL); *n = 1235*	−0.88 (−3.30, 1.54)	−0.90 (−3.35, 1.55)	−0.07 (−2.55, 2.42)	0.72
Males	0.22 (−3.15, 3.60)	1.35 (−2.01, 4.71)	1.15 (−2.18, 4.49)	0.36
Females	−2.02 (−5.39, 1.35)	−2.48 (−5.96, 1.01)	−1.12 (−4.76, 2.53)	0.79
Triglycerides (mg/dL); *n = 1236*	−25.6 (−50.6, −0.6)	−22.7 (−48.0, 2.6)	−29.6 (−55.3, −3.8)	0.10
Males	−18.0 (−48.4, 12.4)	−24.6 (−54.7, 5.5)	−30.8 (−60.7, −1.0)	0.045*
Females	−30.5 (−70.2, 9.4)	−24.4 (−65.6, 16.8)	−26.0 (−69.1, 17.2)	0.51
Fasting glucose (mg/dL); *n = 1234*	5.6 (−4.2, 15.5)	−5.0 (−15.0, 4.9)	−8.5 (−18.6, 1.6)	<0.01*
Males	8.8 (−5.6, 23.2)	−10.9 (−25.2, 3.4)	−12.3 (−26.5, 1.9)	<0.01*
Females	2.7 (−10.8, 16.1)	−2.0 (−15.9, 12.0)	−7.4 (−21.9, 7.2)	0.13
Systolic blood pressure (mmHg); *n = 1235*	−2.2 (−5.4, 1.0)	−0.9 (−4.2, 2.4)	−2.2 (−5.5, 1.1)	0.46
Males	−1.6 (−6.3, 3.1)	−3.8 (−8.5, 0.8)	−3.4 (−8.0, 1.2)	0.11
Females	−2.9 (−7.4, 1.6)	1.3 (−3.3, 6.0)	−1.5 (−6.3, 3.3)	0.74
Diastolic blood pressure (mmHg); *n = 1235*	−1.3 (−2.8, 0.3)	−1.4 (−3.0, 0.2)	−0.9 (−2.5, 0.7)	0.59
Males	−0.9 (−3.4, 1.6)	−2.3 (−4.8, 0.2)	−1.3 (−3.8, 1.1)	0.32
Females	−1.6 (−3.6, 0.4)	−0.6 (−2.7, 1.5)	−0.6 (−2.8, 1.6)	0.74

*$p < 0.05$

[a] Associations assessed using linear mixed effects models with a random intercept for each participant. The reference group for manganese is the first quartile (≤0.33 µg/L). Models are adjusted for baseline values of sex (except in sex-stratified models), age (years), ethnicity (Hispanic, non-Hispanic), annual gross household income (<$10,000, $10,000-$24,999, ≥$25,000), smoking status (<100 cigarettes in lifetime (never), ≥100 cigarettes in lifetime and does not currently smoker (former), ≥100 cigarettes in lifetime and currently smokes (current)), caloric intake (kcal/day), and urinary creatinine (g/L). Values reported are β (95% confidence interval).
